# Single blind randomized Phase III trial to investigate the benefit of a focal lesion ablative microboost in prostate cancer (FLAME-trial): study protocol for a randomized controlled trial

**DOI:** 10.1186/1745-6215-12-255

**Published:** 2011-12-05

**Authors:** Irene M Lips, Uulke A van der Heide, Karin Haustermans, Emile NJT van Lin, Floris Pos, Stefan PG Franken, Alexis NTJ Kotte, Carla H van Gils, Marco van Vulpen

**Affiliations:** 1 Department of Radiation Oncology, University Medical Center Utrecht, Utrecht, The Netherlands; 2 Department of Radiotherapy, The Netherlands Cancer Institute - Antoni van Leeuwenhoek Hospital, Amsterdam, The Netherlands; 3 Department of Radiation Oncology, Leuven Cancer Institute, University Hospital Gasthuisberg, Leuven, Belgium; 4 Department of Radiation Oncology, Radboud University Nijmegen Medical Centre, Nijmegen, The Netherlands; 5 Julius Center for Health Sciences and Primary Care, University Medical Center Utrecht, Utrecht, The Netherlands

**Keywords:** microboost, prostate cancer, external beam radiotherapy, FLAME-trial

## Abstract

**Background:**

The treatment results of external beam radiotherapy for intermediate and high risk prostate cancer patients are insufficient with five-year biochemical relapse rates of approximately 35%. Several randomized trials have shown that dose escalation to the entire prostate improves biochemical disease free survival. However, further dose escalation to the whole gland is limited due to an unacceptable high risk of acute and late toxicity. Moreover, local recurrences often originate at the location of the macroscopic tumor, so boosting the radiation dose at the macroscopic tumor within the prostate might increase local control. A reduction of distant metastases and improved survival can be expected by reducing local failure. The aim of this study is to investigate the benefit of an ablative microboost to the macroscopic tumor within the prostate in patients treated with external beam radiotherapy for prostate cancer.

**Methods/Design:**

The FLAME-trial (**F**ocal **L**esion **A**blative **M**icroboost in prostat**E **cancer) is a single blind randomized controlled phase III trial. We aim to include 566 patients (283 per treatment arm) with intermediate or high risk adenocarcinoma of the prostate who are scheduled for external beam radiotherapy using fiducial markers for position verification. With this number of patients, the expected increase in five-year freedom from biochemical failure rate of 10% can be detected with a power of 80%. Patients allocated to the standard arm receive a dose of 77 Gy in 35 fractions to the entire prostate and patients in the experimental arm receive 77 Gy to the entire prostate and an additional integrated microboost to the macroscopic tumor of 95 Gy in 35 fractions. The secondary outcome measures include treatment-related toxicity, quality of life and disease-specific survival. Furthermore, by localizing the recurrent tumors within the prostate during follow-up and correlating this with the delivered dose, we can obtain accurate dose-effect information for both the macroscopic tumor and subclinical disease in prostate cancer. The rationale, study design and the first 50 patients included are described.

**Trial registration:**

This study is registered at ClinicalTrials.gov: NCT01168479

## Background

Localised prostate cancer can be treated by radical prostatectomy, brachytherapy or external beam radiotherapy. For low risk tumors the results of external beam radiotherapy are comparable to radical prostatectomy and brachytherapy with freedom from biochemical failure rates approximating 95% after 5- to 10-year follow-up [1]. For high risk patients, external beam radiotherapy is preferred. The outcome for intermediate and high risk patients is insufficient with freedom from biochemical failure ranging between 60% and 75% after 5- to 10-years follow-up [2-4].

Several randomized trials have proven that dose escalation in external beam radiotherapy improves the biochemical disease free survival [2,4-6]. Pollack *et al*. [2] compared the efficacy of 70 Gy versus 78 Gy on 305 patients with stage T1-3 prostate cancer. For patients with a pretreatment PSA > 10 ng/mL the freedom from biochemical failure rate at five year was 43% versus 62% respectively, in favor of the higher dose group. The randomized trial from Peeters *et al*. [4] compared 68 Gy versus 78 Gy on 669 patients with stage T1-4 prostate cancer. Five-year freedom from failure rate was significantly improved from 54% to 64%. Further increase in dose is considered to improve the treatment results even further [2,4,7]. Dose response models suggest that tumor areas with severe hypoxia will need very high (ablative) doses [8]. Moreover, local recurrences often occur at the site of the primary macroscopic tumor [9,10], so boosting the radiation dose to the macroscopic tumor might increase the local control. An improvement in distant metastases and survival can be expected by reducing local failure, due to the fact that local failure is associated with distant metastases and mortality [11-13].

Dose escalation to the entire prostate is not considered feasible by reason of unacceptable toxicity risks. This problem can be overcome by partial boosting strategies. In this way, the macroscopic tumor can be irradiated to a very high dose, while the dose constraints to the rectum and bladder can be maintained [14,15]. This approach is being used to deliver a microboost to the dominant tumor region in a number of pilot studies [16-19]. The dose to the macroscopic tumor is increasingly escalated. The highest dose was delivered in a feasibility study by Singh *et al*. [20] who treated 3 patients with an ablative dose of 95 Gy to the macroscopic tumor within the prostate with no severe toxicity (≥ grade 3).

To investigate the benefit of an ablative microboost to the macroscopic tumor within the prostate, we started a randomized controlled trial (the FLAME-trial: **F**ocal **L**esion **A**blative **M**icroboost in prostat**E **cancer, clinical trials: study protocol number NCT01168479). The purpose of this trial is to assess whether a dose escalation to the macroscopic tumor increases the five-year freedom from biochemical failure rate. Furthermore, we will assess the influence of this dose escalation on treatment-related toxicity, quality of life (QoL) and disease-specific survival.

## Methods/Design

### Study design

The FLAME-trial is a multicenter randomized controlled trial. Patients are recruited during the intake consultation at the Department of Radiation Oncology in one of the participating centers. After giving informed consent, patients are randomized either to the standard arm or to the experimental arm. Patients in the standard arm receive radiotherapy according to the current standard [4], namely 77 Gy in 35 fractions of 2.2 Gy to the whole prostate. Patients in the experimental arm receive an additional integrated microboost to the macroscopic tumor to a total dose of 95 Gy in 2.7 Gy fractions.

To ensure unbiased assessment of QoL measurements, the patients are blinded to the actual treatment given (receiving an ablative microboost or not). The treating physician needs to be informed about the actual treatment, to be able to judge the treatment plans. This is unlikely to influence the assessment of the objective primary endpoint of the trial.

### Patients

Men with histological proven intermediate or high risk adenocarcinoma of the prostate, who will receive external beam radiotherapy using optimal position verification with implanted fiducial gold markers, are eligible for the study. Intermediate or high risk is defined according to the currently internationally accepted criteria from Ash *et al*. [21]. Intermediate risk is defined as patients having one factor of stage T2b-c, or Gleason score = 7, or initial prostate-specific antigen (iPSA) = 10-20 ng/mL. Patients having more than one of these factors or having stage T3, or Gleason score >7, or iPSA > 20 ng/mL are defined as high risk. Patients with low risk tumors are not included in this study, because the treatment outcome for this group is already excellent with a 10-year prostate cancer-specific survival approximating 95% [1].

Exclusion criteria are: previous pelvic irradiation, previous prostatectomy, World Health Organization (WHO) performance score > 2 [22] (= symptomatic, >50% during the day in bed, but not bedbound (score 3) or bedbound (score 4)), International Prostate Symptom Score (IPSS) ≥ 20, transurethral resection of the prostate (TURP) within 3 months from start of the treatment, general contraindications for MRI (i.e. cardiac pacemaker, metal implants or history of severe allergic reaction after administration of contrast agent) or the use of anti-coagulants that cannot be discontinued for the gold markers implantation.

The study protocol is approved by the Medical Ethical Committees of the participating hospitals. Written informed consent will be obtained from all patients.

### Randomization

Randomization is performed by an independent trial center. If a patient meets the inclusion criteria and has provided informed consent, the physician contacts the trial center. To prevent randomly occurring differences in important prognostic factors across the two randomized groups, the randomization is stratified by TURP, hormonal treatment and by centre. A TURP prior to radiotherapy is associated with significantly more late genitourinary toxicity [23,24]. The likely mechanism of increased late toxicity is related to the relative devascularisation of the urethra after TURP and the decreased capability of the mucosa to repair sublethal damage after radiotherapy [24]. Hormonal treatment is a prognostic unfavorable factor for late genitourinary side effects [23,25] and erectile impotence [26-28]. Furthermore, a protective effect for hormonal treatment is reported for acute gastrointestinal side effects [23,29,30]. To prevent small numbers of patients in a particular hospital from all receiving, by chance, the same treatment, the hospital is chosen as one of the factors for stratified randomization as well.

### Time schedule

Between October 2009 and October 2010, the first 50 patients were included at the Department of Radiation Oncology of the University Medical Center Utrecht. Based on this accrual and the fact that other participating centers recently started to include patients as well, we expect that the accrual will be completed within 5 years from start.

### Radiotherapy

To minimize the positioning errors during treatment all patients are treated with an online position verification protocol using implanted fiducial gold markers [14,31,32]. Radiotherapy will be delivered with advanced radiotherapy techniques to be able to create adequate dose distributions.

A mean dose of 77 Gy in 35 fraction of 2.2 Gy is prescribed to the entire prostate gland [33,34]. The dose in the part of the PTV overlapping the rectum and bladder is limited to keep the risk of severe gastrointestinal and genitourinary toxicity acceptable.

To provide an accurate delineation of the prostate gland with respect to the surrounding tissues [35,36], the prostate is delineated on a computed tomography (CT) scan combined with a registered magnetic resonance imaging (MRI) scan. The rectum is contoured from the anus or ischial tuberosities to the rectosigmoid flexure or sacroiliac joints. The bladder is completely outlined from the bladder neck to the dome.

The precise execution of the radiotherapy treatment can differ in each participation center, but the treatment will always meet the above mentioned criteria.

### Intervention

Patients randomized to the experimental arm are treated with the current standard of 77 Gy to the whole prostate and in addition receive an integrated microboost to the macroscopic tumor to reach a total dose of 95 Gy in 35 fractions of 2.7 Gy. To delineate the macroscopic tumor within the prostate, different MR imaging techniques are used. In addition to an anatomic T2 weighted sequence, a combination of the following functional imaging modalities can be used [37,38]. Dynamic contrast-enhanced (DCE)-MRI gives a characterization of the tissue vasculature [39]. With this technique it is possible to detect areas with macroscopic tumor, because tumors tend to contain higher density of leaky blood vessels. With diffusion-weighted imaging (DWI)-MRI the mobility of water molecules is measured [40]. Tumor tissue can be identified on DWI-MRI, because in tumor the extracellular volume is reduced, leading to reduced water diffusion in tumor tissue. MR spectroscopic imaging (MRS) provides metabolic information with cancer regions showing higher choline and lower citrate levels [41].

### Primary endpoint

To evaluate whether the addition of an ablative microboost to the macroscopic tumor within the prostate increases the five-year freedom from biochemical failure rate compared to the current standard of care. Biochemical failure is defined according to the Phoenix definition as a PSA rise of 2 ng/mL above the nadir PSA level [42]. The PSA level will be measured every six months until 10 years after treatment.

### Secondary endpoints

Secondary endpoints are treatment-related toxicity, QoL and disease-specific survival. Treatment-related toxicity is measured by the Common Toxicity Criteria for adverse events version 3.0 (CTCAE) [43]. The following adverse events are scored: urinary frequency/urgency, urinary retention, bladder spasms, urinary incontinence, genitourinary hemorrhage, dysuria, rectal or perirectal pain, proctitis, diarrhea, flatulence hemorrhoids, anal incontinence, erectile dysfunction. The physician in attendance scores the complaints before treatment, acute toxicity (weekly during treatment and 4 weeks after treatment) and late toxicity (every six months until 10 years after treatment). All symptoms are registered even if they occur only on one single occasion. Grade > 2 is considered severe toxicity.General health-related QoL is measured using the RAND-36 generic health survey [44], cancer-specific QoL using the European Organization for Research and Treatment of Cancer (EORTC) core questionnaire (QLQ-C30) [45], and the prostate tumor-specific QoL using the EORTC prostate cancer module (QLQ-PR25) [46]. The disease- and treatment-related side effects are only a component of health-related quality of life [47]. To address the components of overall well-being, a general instrument is used in addition to the disease-specific questionnaires [48-50]. The RAND-36 assesses physical and social functioning, physical and emotional role restriction, mental health, vitality, pain, general health and change in health. The EORTC QLQ-C30 contains five functional scales, three symptoms scales, a global QoL scale and six single-items. The EORTC QLQ-PR25 assesses urinary, bowel and sexual symptoms and functioning, and the side effects of hormonal treatment. The first questionnaire is handed over to the patient one week before treatment at the Department of Radiation Oncology and the next questionnaires are sent to the patient every six months until 10 years after the completion of the treatment. It is important to measure QoL every 6 months after treatment, to be able to determine the point in time at which the QoL changes, for example due to side-effects after treatment.

For disease-specific survival, death with metastases is considered a death caused by the disease.

### Safety

An independent data safety monitoring board (DSMB) will evaluate the toxicity and clinical outcome. The DSMB receives an update of the toxicity in total and per treatment arm every 3 months. Serious toxicity, defined as any acute or late toxicity requiring surgical intervention, any grade 4 toxicity, and any not-transient (duration >6 months) late toxicities grade 3, will immediately be reported to the DSMB. An overview of the percentage of biochemical recurrences per treatment arm together with the evaluated number of patients per arm will be sent to the DSMB yearly as well as a statistical comparison of the incidence of serious and less serious toxicities in the two arms. The DSMB decides on stopping or continuing the trial. Exact rules cannot be specified in advance, but an increase in the incidence of toxicity (grade 3-4) with 5% or a smaller, but statistically significant increase, are among the reasons to consider stopping the trial. The DSMB can decide to prematurely stop the trial in case the improvement in the experimental arm is higher than anticipated in the trial design.

### Sample size considerations

The statistical power of the study was calculated for the primary endpoint (five-year freedom from biochemical failure). Based on data of two randomized clinical trials [2,51] reporting the treatment results of patients treated with a radiation dose equal to the dose of the standard arm of our trial, we expect that the five-year freedom from biochemical failure of the standard arm will be approximately 64%. We expect that an additional ablative microboost to the macroscopic tumor will increase this number with at least 10%. A one-sided log rank survival power analysis shows that the length of follow-up after accrual of the last patient should be 3.5 years to detect a difference of 10% (64% and 74% free from biochemical failure after 5 years, in the control arm and experimental arm, respectively) with a power of 80% at a one-sided 5% significance level. This is under the assumption that the patients enter the study during an accrual period of 5 years, 50% of the enrollment is complete when 70% of the accrual time has past and that 20% of the patients in both arms are lost to follow-up during the follow-up period of 5 years. The reason to choose a one sided p-value is that, although extremely improbable, an increase in biochemical failure in the experimental arm would lead to the same action as no difference at all between the two treatment arms. This is because the experimental treatment will only be implemented if it is significantly better than the usual treatment, due to the increased toxicity risk in the experimental arm.

### Data analysis

All analysis will be performed according to the intention-to-treat principle. Cox proportional hazards regression will be used to analyze differences in biochemical failure and disease-specific survival between the two treatment arms. Survival curves will be estimated by the Kaplan-Meier technique. Differences between both groups in the incidence of acute side-effects will be tested with the Chi square test. The incidence of late toxicity will be analyzed actuarially with the Kaplan-Meier method, the log rank test and Cox regression analysis. To analyze differences in QoL between the two treatment groups over the time points, a general linear model repeated-measures analysis of covariance will be performed [52]. A change of 10% (or in general, 0.5 standard deviation) of the scale width is perceptible to patients as a meaningful change [53]. Because of the multiple comparisons for the QoL items, the p-value is set at a conservative 0.01 for determining statistical significance [54]. For all other analyses, which do not include the QoL measures, a p-value of < 0.05 is considered statistically significant.

#### Inclusion of the first 50 patients

The first 50 patients included in the FLAME- trial, were treated between October 2009 and October 2010 at the Department of Radiation Oncology of the UMC Utrecht. All patients received seven-beam intensity-modulated radiotherapy (IMRT). A mean dose of 77 Gy in 35 fraction of 2.2 Gy was prescribed to the planning target volume (PTV) and at least 70 Gy was prescribed to 99% of the PTV [33,34]. The radiation margin around the prostate depends on several uncertainties in the daily clinical practice [55-57]. For our institute based on minimal positioning errors [32] and a simulation of their impact on dose coverage [14], a PTV margin of 4 mm was chosen. We aimed at limiting the dose to the rectum and bladder so that ≤ 5% of the rectum and ≤ 10% of the bladder receives a dose of ≥ 72 Gy. Furthermore, a volume of 1 cc of the bladder and rectum receives a maximum dose of 80 Gy and 77 Gy, respectively, and ≤ 50% of the rectum receives a dose of ≥ 50 Gy [34]. All treatment plans were checked by two investigators (UAH and MV) before start of the treatment. The beam directions were 0º, 50º, 100º, 155º, 205º, 260º and 310º. The location of the fiducial markers was determined by visualizing the markers using portal images of the first segment of the 0º beam and the 260º beam. A difference of more than 1 mm compared to the planning-CT was corrected online. After 5 fractions the average rotation of the prostate was calculated. A rotation of 3º around the anterior-posterior or the left-right axis and a rotation of 6º around the cranio-caudal axis was corrected by changing the gantry or table rotation or the collimator angle. The portal images of the first segment of the remaining beams were used to determine the average intrafraction prostate motion [58]. For each patient the individual intrafraction and remaining rotational errors were used to calculate the actual delivered doses to the target and the organs at risk.

The patients were treated in supine position. One hour before the pretreatment planning scans and the radiotherapy sessions, patients were instructed to drink 500 ml to create a full bladder. A full bladder during radiotherapy results in a decreased amount of bladder volume in the high dose region and a lower dose to bowel loops compared to treatment with an empty bladder [59]. No antiflatulent diet or laxative was prescribed [60]. When the rectum filling on CT and MRI differed considerably, a new CT or MRI scan was performed, to minimize the registration uncertainty between these two imaging modalities.

**Figure 1:**
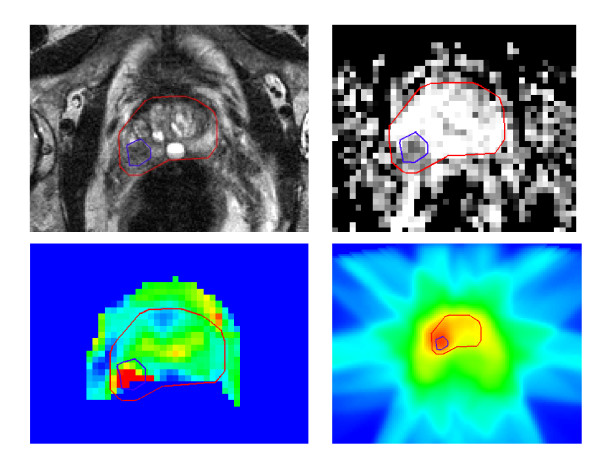
Example of the delineation of the macroscopic tumor area (GTV) on T2 weighted MRI (a), an apparent diffusion coefficient map derived from diffusion-weighted MRI (b) and a Ktrans parameter map obtained from dynamic contrast-enhanced MRI (c) and the dose distribution (d).

For delineation of the macroscopic tumor within the prostate, defined as the gross tumor volume (GTV), anatomical and functional imaging was performed on a 3 Tesla MRI scanner (Achieva Philips Medical Systems, Best, the Netherlands). The exam included 3 anatomical scans: a multislice T2 weighted turbo spin echo (TSE) sequence (TR/TE 8400/120 ms), a T1 weighted sequence and a balanced turbo field echo (TFE) sequence (TR/TE 2.8/1.4 ms, FOV = 25 cm, slice thickness = 1 mm). The DCE-MRI protocol consists of a 3D spoiled gradient echo sequence (TR/TE 4.0/1.0 ms, flip angle 6º). Scans were repeated 120 times at 2.4 s interval. A single acquisition consisted of 20 axial slices of 2.5 mm. The field of view was 40 × 40 cm^2^, the reconstruction matrix 160 × 160. For contrast enhancement, 0.1 mg/kg body weight gadobutrol (1.0 M (Gadovist, Schering) was injected intraveneously. Trace-kinetics modeling was done using the Tofts model [61] resulting in 3D maps of the transfer constant K^trans^. Diffusion-weighted imaging scans were performed using a multislice single shot SE-EPI sequence (FOV = 38 cm, slice thickness = 3 mm, intersection gap = 1 mm, TR/TE = 5000/54 ms, acquisition matrix = 152 × 107, b values = 0, 300, 5000, 100 s/mm^2^). The delineation of the GTV was done by the treating physician and checked by two investigators (UAH and MV) before start of the treatment. To account for possible extracapsular extension, the delineation of the macroscopic tumor was expanded with an extra margin of 4 mm [62]. Figure 1 shows an example of the delineation of the GTV and the dose distribution for a patient in the experimental arm. Table 1 shows the patient characteristics of the first 50 patients. Twenty-three patients were randomized into the experimental arm and 27 into the standard arm.

**Table 1:** Patient characteristics

	Patients included in the FLAME-trial (*n *= 50)	
	
Characteristic	**No**.	%
Age, years		
Median	70.5	
Upper and lower quartile	66.8 - 73.0	
Tumor stage		
T1	3	6
T2	8	16
T3	38	76
T4	1	2
Tumor grade		
Gleason score 4-6	14	28
Gleason score 7	17	34
Gleason score 8-10	19	38
iPSA, ng/mL		
Median	14.3	
Upper and lower quartile	9.9 - 21.0	
Cardiovascular disease	33	66
Diabetes mellitus	5	10
Prescription of long term hormonal therapy	26	52
History of TURP	10	20

*Abbreviations*: TURP = transurethral resection of the prostate; iPSA = initial prostate-specific antigen.

## Discussion

The FLAME-trial is designed to investigate the effect of an ablative microboost to the macroscopic tumor for patients treated with external beam radiotherapy for prostate cancer.

Previous studies demonstrated that the rate of toxicity after high dose external beam radiotherapy with the use of accurate position verification is low and consequently high QoL is reported [34,63-66]. Planning studies showed that an ablative microboost to the macroscopic tumor was theoretically feasible within the currently used dose constraints for rectum and bladder [15,67,68]. Furthermore, a feasibility study of Singh *et al*. [20] reported excellent early toxicity after simultaneous integrated IMRT boost of 95 Gy to the intraprostatic lesions. As a result, with the use of optimal position verification combined with the currently used dose constraints, the toxicity in the experimental treatment arm with the ablative microboost of 95 Gy is expected to be acceptable.

Previous trials demonstrated a biochemical benefit of dose escalation. However, up to now none of the dose escalation trials were able to detect an improvement in disease specific or overall survival. However, all trials were designed for biochemical survival instead of overall or disease-specific survival due to the natural behavior of prostate cancer. For this reason the FLAME-trial is also powered for biochemical disease free survival. An improvement in local control without a proven benefit in overall survival is only acceptable when severe toxicity remains limited. Furthermore, to establish whether a benefit in biochemical failure free survival also counterbalances the negative aspects of dose escalation, such as a small increase in toxicity, it is important that QoL is taken into account. Therefore, repeated QoL measurements are performed in patients included in this trial.

The precise delineation of the macroscopic tumor within the prostate is a topic of ongoing research [37,38,69]. In the FLAME-trial the delineation of the macroscopic tumor is based on anatomical and functional imaging according to the current opinion. The different imaging techniques might show conflicting results about the boundaries of the macroscopic tumor area, leading to difficult delineation decisions. Therefore, it is of major importance to investigate the precise location of a recurrence and to establish what dose was prescribed to that location. When a patient shows a rising PSA without distant metastases, DCE-MRI and MRS can be used to detect the location of recurrent prostate cancer [70-74]. By correlating the dose distribution of the initial radiotherapy with the location of a local recurrence, accurate dose-effect information can be obtained. The dose-effect data generated from this analysis will help us to evaluate the required dose for each cancer subunit and to provide a better understanding of the different imaging techniques [38]. The dose distributions of the patients treated in the experimental arm, are inhomogeneous with very low and very high delivered doses, and for that reason provide important dose-effect information to create a reliable dose-effect curve.

A randomized study design is indicated to resolve the problem of confounding effects. To our knowledge, no other randomized controlled trials are being performed to investigate the benefit of an ablative microboost to the macroscopic tumor in prostate cancer patients. Beside Singh *et al*. [20], three other groups performed a pilot study in which a microboost to the dominant tumor region was delivered. Miralbell, *et al*. [16] treated 50 patients, after 64-64.4 Gy in 1.8-2 Gy fractions to the whole prostate, with a hypofractionated boost of 2 fractions of 5 to 8 Gy to the dominant tumor region, delineated by anatomical imaging. After a median follow up time of 63 months, a 5-year biochemical disease-free and disease-specific survival of 98% and 100%, respectively, were reported with acceptable long-term toxicity. Gaudet *et al*. [17] selectively delivered a brachytherapy hyperdosage of ≥ 216 Gy (150% of the prescribed dose) to the macroscopic tumor, defined according to positive areas on sextant biopsy, in 70 patients with localized prostate cancer treated with permanent seed prostate implant. No difference in acute or late toxicities compared to 120 patients with a standard plan were seen. Fonteyne *et al*. and De Meerleer *et al*. [18,19] performed the largest trial in which 230 patients were treated with a mean dose of 81-82 Gy to a dominant lesion, defined by T2 weighted MRI or MRI plus spectroscopy. With the use of IMRT and daily ultra-sound based prostate positioning, the acute toxicity remained low with no grade 3 or 4 acute gastrointestinal toxicity and 7% grade 3 genitourinary toxicity.

Analyses of the actual delivered dose in the first 50 patients included in the FLAME-trial, revealed that it is possible to deliver a high dose to the macroscopic tumor area without compromising the dose constraints for the nearby organs at risk. The influence of the remaining intrafraction and rotational errors using an online position verification protocol is minimal.

In conclusion, the aim of the FLAME-trial is to assess, in patients treated with external beam radiotherapy for prostate cancer, the potential benefit of an additional ablative microboost to the macroscopic tumor on biochemical control. In addition, the subgroup of patients that will develop a local recurrence within the prostate after treatment can be used to obtain accurate dose-effect information for both the dominant lesion and subclinical disease in prostate cancer.

The study protocol was approved by the Medical Ethical Committee. The trial is registered at http://ClinicalTrials.gov (Registration identification number: NCT01168479; URL: http://clinicaltrials.gov/ct2/show/NCT01168479)

## List of abbreviations used

CT: Computed Tomography; CTCAE: Common Toxicity Criteria for adverse events; DCE: dynamic contrast-enhanced; DSMB: data safety monitoring board; DWI: Diffusion-weighted imaging; EORTC QLQ-C30: European Organization for Research and Treatment of Cancer core questionnaire; EORTC QLQ-PR25: EORTC prostate cancer module; GTV: gross tumor volume; IMRT: intensity-modulated radiotherapy; IPSS: International Prostate Symptom Score; MRI: Magnetic Resonance Imaging; iPSA: initial prostate-specific antigen; PTV: planning target volume; QoL: quality of life; TURP: transurethral resection of the prostate; WHO: world health organization.

## Competing interests

The authors declare that they have no competing interests.

## Authors' contributions

IML participated in the design and data collection and drafted the manuscript. UAH participated in the study concept and design and revised the manuscript critically. ANTJK carried out the dose reconstruction. SPGF, KH, FP and ENL critically revised the manuscript CHG participated in the study design and performed the statistical calculations. MV participated in the study concept and its design and coordination and helped to draft the manuscript. All authors read and approved the final manuscript.
